# The Role of Myofunctional Therapy in Treating Sleep-Disordered Breathing: A State-of-the-Art Review

**DOI:** 10.3390/ijerph18147291

**Published:** 2021-07-08

**Authors:** Marina Carrasco-Llatas, Carlos O’Connor-Reina, Christian Calvo-Henríquez

**Affiliations:** 1 Department of Otolaryngology, Hospital Universitario Dr. Peset, 46017 Valencia, Spain; 2 Department of Otolaryngology, Hospital QuironSalud Marbella, 29603 Marbella, Spain; carlos.oconnor@quironsalud.es; 3 Rhinology Study Group of the Young-Otolaryngologists of the International Federations of Otorhinolaryngological Societies (YO-IFOS), 13005 Marseille, France; christian.calvo.henriquez@gmail.com; 4 Service of Otolaryngology, Hospital Complex of Santiago de Compostela, 15706 Santiago de Compostela, Spain

**Keywords:** sleep apnoea, obstructive, myofunctional therapy, snoring

## Abstract

Myofunctional therapy (MFT) may have a role in improving muscle tone and alleviating upper airway collapse in sleep-disordered breathing. The purposes of this state-of-the-art review are to first review systematically the current literature on the effectiveness of MFT in treating sleep-disordered breathing and then to provide an overview of the current understanding of patient selection, side effects, type and duration of exercises, guidance of exercise performance, evaluation of results, and how best to promote adherence. PubMed (Medline), the Cochrane Library, and the EMBASE, Scopus and SciELO databases were checked for relevant studies by three authors, and a total of 23 studies were included. This review focuses only on adults with sleep-disordered breathing. The available evidence shows a positive effect of MFT in reducing sleep apnoea, as measured using polysomnography and clinical variables (including snoring). There is no evidence of the utility of MFT for treating upper airway resistance syndrome, the duration of the effects of MFT, or regarding which MFT protocol is best. Despite these knowledge gaps, the available evidence suggests that MFT is a safe treatment modality.

## 1. Introduction

Sleep-disordered breathing (SDB) is a common disease whose prevalence varies between countries and studies, but has been reported to be as high as 49.7% of men and 23.4% of women. SDB is related to poor health outcomes, increasing health costs and poor quality of life [1]. According to Eckert et al. [2], the physiopathology of SDB involves a weak muscular response in many patients; therefore, therapies to address this factor may have a role in the treatment of SDB.

Active training of the oropharyngeal muscles as a treatment modality for sleep disordered breathing (SDB) was first reported in 2006 by Puhan et al. [3], and interest in this treatment modality has increased since then. Such training is commonly included under the concept of myofunctional therapy (MFT). Interest in the potential use of MFT is based on reports of the dysfunction and hypotony of the orofacial and pharyngeal muscles as contributors to airway collapse in SDB.

Despite the increasing interest in and potential benefits of this treatment modality, several major problems have arisen. First is the concept of MFT, which is based on the principles of speech language pathology initially designed to address speech and swallowing disorders and oral growth. MFT comprises primarily a series of exercises designed to improve tongue position and function, the lip seal, and nasal breathing. However, the number of treatments included under MFT has increased to also include breathing exercises and orthopaedic devices, but these are not, strictly speaking, MFT. Although this wide application of the MFT concept may not be wrong, differences in the conceptualization of MFT make it difficult for professionals to find a common approach and to draw solid conclusions from the available evidence.

The second major problem is the lack of objective diagnostic tools. Because the diagnosis of myofunctional disorders relies mainly on physical examination, different professionals may provide different diagnoses. Third, SDB is not yet recognized as a myofunctional problem and, despite the potential benefits of MFT, its purpose is not the treatment of SDB. As a consequence, MFT is not covered by medical insurance providers as a treatment for SDB.

These problems affect the application of the therapy. Only by developing clear definitions, objective measurements, and strong scientific evidence will this treatment modality find a place in the standard treatment of SDB. The purposes of this state-of-the-art review are to first provide a systematic overview of the current literature on the effectiveness of MFT as a treatment for SDB, and then to describe the current understanding of patient selection, side effects, type and duration of exercises, guidance of exercise performance, evaluation of results, and how best to promote adherence. This review focuses only on adults with SDB.

## 2. Materials and Methods

### 2.1. Literature Search: Inclusion and Exclusion Criteria

The criteria for considering studies for this systematic review were based on the population, intervention, comparison, outcome (PICO) framework [4].

- Population: Adults experiencing SDB, including snoring, upper airway resistance syndrome, or sleep apnoea.
- Intervention: Any type of MFT, although following Camacho et al. [5], we did not consider the range of MFT to include singing exercises or playing wind instruments.
- Comparison: From before to after MFT in uncontrolled studies (quasi experimental studies) or between intervention and no-intervention cohorts in controlled studies (cohort and clinical trials).
- Outcome: Six research questions were defined. (1) Is MFT useful for treating SDB? (2) Which patients benefit most from MFT? (3) What are the secondary effects of MFT? (4) Which type of MFT is best? (5) How should the effects of MFT be assessed? (6) How long do the effects of MFT last?
- Types of studies: Clinical trials, case series, and prospective and retrospective cohort studies published in peer-reviewed journals. We did not include case reports, theses, narrative reviews, or meeting communications. There were no restrictions by date or publication type. The search was last updated in February 2021. We included studies published in English, Spanish, German, French, Italian, or Portuguese.
- Exclusion criteria: (1) Studies that included syndromic patients, (2) duplicated publications, (3) studies in which the individual effect of MFT was not explored, (4) studies that did not answer any of the research questions, and (5) studies that included both adults and children without subgroup analysis.

### 2.2. Search Strategy

We followed the recommendations of the PRISMA statement [6] to perform a systematic review and searched the following databases: PubMed (Medline), the Cochrane Library, EMBASE, Scopus, and SciELO. We used a predefined search strategy that is described in Table S1. The abstracts of the retrieved papers were reviewed thoroughly by three authors (C.C.-H., M.C.-L., and C.O.-R.), and publications that could potentially fulfil the inclusion criteria were selected for a full-text review. In case of discrepancies between reviewers regarding the selection of the abstracts, the corresponding papers were included in the full-text review stage for the final assessment. We also manually reviewed the references of all of the selected articles to identify any potentially missing publications.

### 2.3. Data Extraction and Analysis

Three authors (C.C.-H., M.C.-L., and C.O.-R.) independently analysed the articles that met the inclusion criteria and extracted the relevant data.

### 2.4. Level of Evidence

The level of evidence was classified according to the Oxford Centre for Evidence-Based Medicine 2011 Levels of Evidence [7].

### 2.5. Statistical Analysis

Data were analysed using STATA for Macintosh v. 15.1 (StataCorp^®^. Los Angeles, CA, USA). No comparison tests were used. Data are presented with 95% confidence intervals (CIs) when possible.

## 3. Results

### Search Results

**Figure 1:**
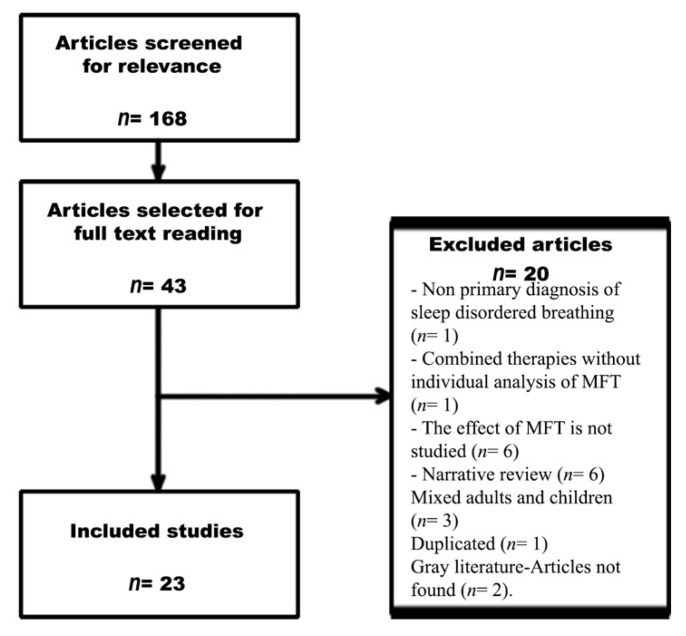
PRISMA flow chart.

A flow chart of the search process is shown in Figure 1. The initial search retrieved 168 publications. After reading all of the titles and abstracts, 43 studies were selected for full reading. A total of 23 studies (411 adult patients and 146 adult controls, excluding data from reviews) met the inclusion criteria.

Of the selected papers, 20 were excluded for the following reasons: non-primary diagnosis of SDB (*n* = 1), combined therapies without individual analysis of MFT (*n* = 1), no analysis of the effects of MFT (*n* = 6), narrative review (*n* = 6), only children included (9), adults and children included but not analysed separately (*n* = 3), duplication (*n* = 1), and grey literature or article not found (*n* = 2).

The search strategy identified 23 studies that met the inclusion criteria [5,8,9,10,11,12,13,14,15,16,17,18,19,20,21,22,23,24,25,26,27,28,29]. The mean sample size (excluding systematic reviews and meta-analyses) was 38.28. The mean age pondered by the sample size and excluding systematic reviews and meta-analyses was 51.19 years. The search strategy retrieved three meta-analyses [5,10,15], one systematic review [23], nine randomized clinical trials [8,11,12,13,19,21,24,25,26], seven quasi-experimental studies [9,16,17,18,20,22,29], one cohort study [14], one case series [27], and no case–control studies.

Table 1 summarizes the available evidence for each research question.

## 4. Discussion

The literature review reflects the increasing interest in the use of MFT for the treatment of SDB, because most of the selected studies were published in the last 10 years. We identified previous systematic reviews, which we have summarized and included in this study. However, these reviews focused on specific questions. This is the first state-of-the-art review that aimed to summarize and critically assess all of the available evidence regarding the role of MFT in the treatment of SDB and to provide evidence-based guidance to clinicians for their daily practice.

The available evidence and the recommendations are summarized in Table 1, and individual data from the selected studies are summarized in Table S2. Specific MFT programmes from the selected studies are summarized in Table S3.

### 4.1. Question 1a: Is MFT Useful for Treating SDB—OSA?

#### 4.1.1. Polysomnographic Variables

Polysomnographic (PSG) variables were included in 18 studies [5,9,10,11,12,13,15,16,17,19,21,22,23,24,25,28,29], including two meta-analyses [5,10] and two systematic reviews [23,28]. We note that the meta-analysis of Hsu et al. [10] and the review by Valbuza et al. [23] reported studies using MFT and respiratory exercises, as well as protocols that included singing exercises and playing wind instruments, which were not considered to be MFT in the meta-analysis of Camacho et al. [5] or in our review.

The primary outcome was the apnoea–hypopnoea index (AHI), measured as events per hour during a sleep study. Secondary PSG outcomes included minimum and average oxygen saturation (mean SaO_2_).

Hsu et al. included five single-blinded randomized controlled trials (RCTs) and four non-blinded RCTs in their meta-analysis. In their analyses of the studies of adults, the average decrease in the AHI was −36.0% ± 25.6% in the intervention groups and −0.3% ± 15.3% in the control groups [10].

The second meta-analysis, by Camacho et al., explored 11 articles, 9 of which included adults [5]. In the adult studies in which MFT was performed for at least 3 months, the decrease in the mean AHI was greater than that reported by Hsu et al. [10]. After excluding articles because of heterogeneity, Camacho et al. found that this variable decreased by 50% from 25.2 ± 14.6/h to 12.6 ± 12.2/h. The oxygen desaturation index was reported by one study as a reduction from 14.53 ± 5.04 before to 9.27 ± 4.27 after MFT [29].

The studies by Verma et al. [16], Erturk et al. [11], and Randerath et al. [25] were the only selected studies that did not find significant differences in the AHI. Verma et al. reported a small difference in the AHI from 20.1 to 19.7 after 3 months of MFT [16], and that other PSG variables improved more. Their results cannot be explained by the contribution of the severity of apnoea to the AHI, because it was similar to that in the other selected papers. A possible explanation is that their training programme, protocol, or patient selection was not described fully. Erturk et al. reported a change in the AHI from 42.6 to 39.9 with MFT [11]. The authors suggested that this improvement may have been related to the severity of the baseline AHI. However, they did not perform subgroup analysis to confirm this. Finally, Randerath et al. used passive therapy with electrical stimulation as MFT [25]. Their treatment protocol was effective only for snoring and did not affect obstructive sleep apnoea (OSA), possibly because only the tongue was stimulated in their treatment protocol.

No articles reported on the comparison between the AHI during rapid eye movement (REM) and non-REM sleep. Therefore, it is not possible to determine whether MFT is helpful in decreasing the severity of OSA during REM sleep, when the muscle tone is lower, or whether it is more useful for decreasing the severity of OSA during non-REM sleep. More studies are needed to address these questions.

#### 4.1.2. Clinical Variables

In this review, we obtained data for a wide spectrum of clinical variables. The most studied variables were the Epworth Sleepiness Scale (ESS) [5,8,9,10,11,12,13,15,16,17,19,23,24,25,27] and the Pittsburgh Sleep Quality Index (PSQI) [8,10,11,12,13,19,23,24].

Although the ESS has been criticized for its low sensitivity, specificity, and predictive values, almost all of the selected papers used it to assess the effects of treatment on daytime sleepiness. According to Hsu et al. [10], five studies that explored the change in the ESS score could be combined in their meta-analysis. They found no statistically significant improvement in ESS score from the baseline (*p* = 0.06), despite a positive average improvement of –2.5 in the intervention groups compared with the controls. However, this small difference may not be clinically relevant, as it has been previously suggested that the minimum clinically relevant difference in the ESS score is four points [30]. By contrast, the meta-analysis of Camacho et al. [5], which included 75 adults, found a statistically significant mean decrease in the ESS score from 14.8 ± 3.5 to 8.2 ± 4.1. This difference between the two meta-analyses may be explained by the exclusion by Camacho et al. of some papers because of heterogeneity, as well as other studies that included respiratory exercises, singing, or playing wind instruments.

The PSQI measures sleep quality on a scale of 0–21 and has been used in eight studies. The meta-analysis of Hsu et al. [10] included four studies that used the PSQI before and after treatment. Overall, there was a statistically significant improvement in PSQI of –1.3 in the intervention group compared with the controls (95% CI –2.4 to –0.2). However, the minimal clinically important improvement in PSQI was –3, and this difference may not be clinically significant. The post-treatment PSQI improved the most in the study that involved lower airway inspiratory muscle therapy compared with the two studies involving lower airway expiratory muscle therapy and one study involving the upper airway. One study that was not included in the meta-analysis of Hsu et al. is the clinical trial reported by Diaferia et al. [21]. They found that the quality of sleep, assessed using the PSQI, improved similarly after treatment with MFT alone or when combined with continuous positive airway pressure (CPAP). However, no effect was observed in the group treated with CPAP alone, which seems to be questionable given the available evidence.

The morning headache symptom was investigated in two studies [16,29], only one of which detected a reduction from 60% to 20% in the number of patients with this complaint [29].

Another positive contribution of MFT is that it seems to favour adherence to the use of CPAP, which has been studied by only one group, Diaferia et al., in a single blinded RCT [21]. Patients in the combined MFT plus CPAP group spent more time using the CPAP device than did those in the CPAP-only group; these effects were observed after both 1 and 3 months. This study also assessed the adherence to CPAP, based on the number of days used for >4 h/night, and reported that adherence was 30% in the CPAP-only group vs. 50% in the combined group. However, solid conclusions cannot be assumed from their data, as this study had an important bias in that the patients in the CPAP-only group were unsupervised, whereas the CPAP plus MFT group had weekly visits with the therapist.

Another possible benefit of MFT is the decrease in cervical perimeter, which has been assessed in seven studies [11,16,19,20,24,25,29] and one systematic review [28]. Five of these studies reported a significant reduction in the cervical perimeter after MFT, although Erturk et al. [11] and Matsumura et al. [20] did not find significant differences. It is noteworthy that Erturk et al. included breathing exercises and Matsumura et al. had a small sample size (11 participants), which could explain these differences between studies.

### 4.2. Question 1b: Is MFT Useful for Treating SDB?—Snoring

Five clinical trials [11,12,19,24,26], two meta-analyses [5,15], and one systematic review [28] have explored the role of MFT in reducing snoring. Even though almost all studies of SDB have included snoring as a variable, only nine studies explored it objectively [5,9,12,15,17,19,25,26,29]. In addition, the objective and subjective methodologies of reporting snoring were heterogeneous, rendering a comparison between the articles challenging.

Camacho et al. performed a meta-analysis to explore specifically the effects of MFT on snoring [15]. They included four studies and reported three main findings. First, snoring improved by about 50% after MFT when assessed using a visual analogue scale (VAS). Second, improvements were seen in all of the study measures (Berlin questionnaires, VAS, and snoring during the sleep study). Third, there was objective improvement in snoring based on PSG variables, with a 31% decrease in the percentage of time spent snoring.

Four RCTs have included snoring as their primary endpoint [19,22,25,26]. Ieto et al. reported that snoring, which was assessed objectively using the snore index, and subjectively by the bed partner, was reduced after MFT compared with the control group [19].

In a clinical trial, Erturk et al. explored snoring subjectively using the Berlin questionnaire [11]. They reported a larger decrease in snoring in patients with severe OSA compared with mild OSA, and in patients with moderate OSA compared with mild OSA.

### 4.3. Question 1c: Is MFT Useful for Treating SDB?—Upper Airway Resistance Syndrome

We could not identify any evidence of the role of MFT in treating upper airway resistance syndrome. We found only one case report [16], which was not included in this review because of its design.

### 4.4. Question 2: Which Patients Benefit Most from MFT?

None of the selected studies differentiated between strength/tonus problems from functional problems in their patient selection. We suggest that these reflect different patient profiles that should be treated differently. We propose that SDB patients can be classified into three groups: one group with conventional oro-myofunctional disorders (tongue protrusion, tongue thrusting, etc.), the second with upper airway muscle hypotony, and the third with both disorders. These differences may explain some of the heterogeneity of the results reported in the literature.

There is scarce information about which patients benefit the most from MFT. Most available evidence comes from scattered data in studies that were not designed to study this variable.

#### 4.4.1. Tongue and Lip Strength

Tongue strength is lower in some patients with sleep apnoea [31], and the effect of MFT is closely related to the increase in tongue strength [9]. Three studies [9,13,22] have shown, using objective assessment, that the improvement in tongue and lip strength after MFT correlates significantly with the improvement in PSG variables. However, despite the biological plausibility that patients with hypotonic musculature would obtain the most benefit from MFT, none of the identified papers examined tongue and lip strength as a selection criterion.

#### 4.4.2. Lingual Fraenulum

Even though most of the identified MFT protocols include tongue mobility exercises based on the belief that a short lingual fraenulum might impair these movements, only one study considered the lingual fraenulum as an exclusion criterion [13].

#### 4.4.3. Severity

Seven studies in adults excluded patients with severe apnoea [8,13,16,18,19,22,24]. The study by O’Connor-Reina et al. [13] is the only one to have included severe cases, and the authors reported a significant improvement after MFT. Therefore, at present, there is no evidence to support the exclusion of patients with severe SDB.

Only one study examined the role of MFT according to the severity of apnoea. Mohamed et al. [17] included two cohorts—one with moderate and the other with severe OSA—and found an improvement after MFT in the cohort with moderate but not severe OSA. By contrast, other authors have suggested that patients with severe SDB are most likely to benefit from MFT. Ieto et al. [19] reported a decrease in the AHI in the moderate OSA group, but not in the whole group of patients. In their RCT, Erturk et al. [11] found a larger decrease in snoring in patients with severe compared with those with mild OSA.

We did not include the systematic review of de Felício et al. [32] in our review, because it combined children and adults, without a separate statistical analysis. They suggested that sleepy patients (mean baseline ESS score of 12 ± 2.6 to 15.4 ± 2.3) showed a significant improvement after MFT. However, no change was observed in the group of patients with median ESS scores of 3–11. It was suggested that patients with more severe ESS scores were those who obtained more significant improvements in symptoms after MFT.

#### 4.4.4. Malocclusion and Craniofacial Anomalies

Eleven studies excluded participants with craniofacial anomalies [9,12,13,16,17,18,19,20,22,24,29]. However, none of them clearly defined craniofacial anomalies or compared the differences in the changes after MFT between patients with and without malocclusion.

#### 4.4.5. Body Mass Index

Ten studies excluded participants with a high body mass index (BMI) [9,11,13,16,17,18,19,21,24,29]. We did not include the paper by Engelke et al. [33] in this review because they combined data for children and adults. However, their subgroup analysis according to BMI showed worse results in snoring patients with obesity according to BMI than in those with a normal BMI. Although the accepted opinion is that people with obesity who snore are less likely to benefit from MFT, there is no scientific evidence to support this opinion.

#### 4.4.6. Positional Apnoea

Suzuki et al. reported a larger decrease in the AHI during sleep in the lateral position compared with the supine position after MFT [9].

#### 4.4.7. Nasal Patency

Thirteen papers excluded patients with impaired nasal breathing [8,9,11,12,13,16,17,18,19,20,21,24,29]. However, none of the identified papers evaluated the predictive value of nasal patency.

#### 4.4.8. Tonsillar Hypertrophy

No studies have examined whether MFT has different effects in patients with or without tonsillar hypertrophy. Five papers excluded this subgroup of patients [13,17,19,21,29].

#### 4.4.9. Scales

Currently, there are no validated scales to identify which subsets of patients with SDB benefit the most from MFT. Folha et al. validated the Expanded Protocol of Orofacial Myofunctional Evaluation with Scores [34], a clinical scale designed to assess facial, masticatory, and upper airway musculature systematically. They found that this instrument seemed to differentiate between patients with and without sleep apnoea, and had good interobserver agreement.

#### 4.4.10. Age, Sex, and Tongue Volume

No studies have examined whether there are differences in the responses to MFT according to age, sex, or tongue volume.

### 4.5. Question 3: What Are the Secondary Effects of MFT?

Overall, MFT seems to be a safe treatment and has a low incidence of minor complications. Diaferia et al. [21] reported “very few side effects”, although they did not provide details of these side effects. Randerath et al. [25] performed an RCT of patients undergoing passive MFT with electrical stimulation of the tongue. The experimental group of patients reported higher rates of erythema, skin irritation, and facial pain compared with the placebo group. In a quasi-experimental study of patients following a mobile app-guided MFT programme, O’Connor-Reina et al. [13] reported one case each of tongue irritation and temporomandibular joint disorder, and three cases of fatigue, which led to patients’ rejection of the use of the app.

### 4.6. Question 4: Which Type of MFT Is Best?

Although MFT seems to have a beneficial role in treating SDB, it is less clear which treatment protocol should be used. For example, the effectiveness of the following factors has not been established: home-based vs. supervised exercises, duration of the programme, and passive vs. active exercises. All of the available reviews and meta-analyses have mixed data by assuming that all techniques and protocols falling under the category of MFT are equally effective, which is probably wrong.

Table S3 summarizes the different protocols described by the authors who reported them. Most of the authors of the papers reviewed adopted a set of exercises to cover the various oropharyngeal structures (tongue, palate, lateral pharyngeal walls, or epiglottis) that, separately or in combination, are involved in the collapse of the pharyngeal airway. The most frequently studied protocol is the one reported by Guimarães et al. [24], which was reproduced and adapted by six authors [8,11,18,20,21,29]. Despite its widespread use by different authors, it has not been compared against other protocols.

It is noteworthy that none of the selected authors focused on proprioceptive training, but instead focused on co-ordination, strength, and mobility mainly by prescribing isometric and isotonic exercises. Only Verma et al. compared three different sets of oropharyngeal exercises and concluded that there were no differences between the programmes [16]. However, their design had important flaws that prevented us from drawing sound conclusions. First, the effects of training were progressive, and the highest percentage in improvement of symptoms achieved by patients in the last phase of treatment compared with the previous two phases did not indicate which set of exercises were most effective. Second, the patients were not randomized and, third, the sample size was small.

Only one study in adults has compared a programme performed under professional supervision (nurse practitioner) with a home-guided programme performed using videotapes and images [12]. The authors found a greater reduction in the AHI and ESS in the experimental group, and concluded that MFT should be tailored to the needs of each patient.

No other studies have compared different treatment protocols. According to the meta-analysis of Camacho et al., despite the heterogeneity, the improvements in PSG outcomes and sleepiness are consistent [15]. Their data suggest that different treatment protocols may produce similar results.

We found no studies comparing the duration of MFT. The shortest treatment protocol was as little as 2 months [25] and the longest was 6 months [9].

Finally, we found no studies comparing active and passive MFT. Although adherence appears to be higher with passive MFT, no firm conclusions can be drawn without comparative studies.

### 4.7. Question 5: How Should the Effects of MFT Be Assessed?

All of the selected studies reported patient adherence to MFT, and most assessed adherence using patient diaries. The mean adherence adjusted by sample size was 79.91%; the highest was 100% [8,9] and the lowest 50% [26].

In their clinical trial, Hsu et al. demonstrated that good compliance was related to a significant improvement in the AHI [10]. Therefore, compliance is an important variable that should be included in any analysis of the technique or programme. Most published reports did not follow an intention-to-treat analysis and did not include patients lost to follow-up. Only two studies, Kim et al. [12] and Ieto et al. [19], followed an intention-to-treat analysis. Kim et al. [12] found significant differences in the AHI, but these were lower than those in previous reports, which may reflect their use of the stricter research protocol.

It is noteworthy that compliance may be lower in patients who have not been studied, as it is well known that the mere observation of patients increases treatment compliance (i.e., the Hawthorne effect). Another important point is that compliance tends to decrease with time after follow-up.

In the study by Kim et al. [12], who compared a cohort of participants following an at-home programme with others following a programme with a nurse practitioner, adherence was higher in the latter group than in the former (82.06% and 72.52%, respectively).

It is noteworthy that the high adherence in patients using a mobile app-guided MFT programme reached a compliance rate as high as 90% [13]. However, another study that used a mobile app reported an adherence as low as 50% [26]. This lower percentage may reflect the lack of option for patients to record their snoring, and they were considered non-adherent to the therapy. More studies are required, but the feedback received by the patient may be important for increasing compliance.

### 4.8. Question 6: How Long Does the Effect Last?

Based on their experience, Guimarães et al. [24] suggested that patients need to exercise the upper airway muscles continuously. However, there is virtually no evidence favouring or rejecting this suggestion. According to the review by Camacho et al. [5], there are limited cases of long-term follow-up for more than 6 months.

Additional concerns arise about the maintenance of the effects after the discontinuation of MFT. Most of the selected authors reported the effects after MFT treatment, but not after discontinuation. To date, the longest follow-up after the discontinuation of MFT was reported by Diaferia et al. [21] as 3 months. Goswami et al. also reported a follow-up of 3–6 months, but they did not report their mean follow-up [26].

## 5. Conclusions

The available evidence demonstrates a positive effect of MFT in reducing sleep apnoea, as shown by the measurements of PSG and the clinical variables in adults. The evidence is solid for snoring reduction measured objectively with PSG variables or subjectively with scales. By contrast, there is no evidence to support the use of MFT in the treatment of upper airway resistance syndrome, nor is there conclusive evidence about the duration of the positive effects of MFT or which MFT protocol is best. Despite these knowledge gaps, the available evidence indicates that MFT is safe and has few and mild secondary effects.

Given the available evidence and the safety of MFT, we suggest that MFT should be initially offered as a non-invasive therapy to patients experiencing SDB. We encourage continued research into this promising treatment modality in order to better understand the optimal methods to select individual patients, exercises, and protocols.

## Figures and Tables

**Table 1:** Summary of the evidence.

Question	Available Evidence	Level of Evidence	Conclusions
Is MFT useful for treating SDB? (PSG variables)	2 meta-analyses [5,10], 2 systematic reviews [23,28], 7 RCTs [11,12,14,19,21,24,25], 1 cohort [14], 5 quasi-experimental [9,16,17,22,29], and 1 case series [27]	Level 1a	Available evidence demonstrates a positive effect of MFT in reducing sleep apnoea as measured by PSG variables.
Is MFT useful for treating SDB? (sleepiness and QoL)	2 meta-analyses [5,10], 2 systematic reviews [23,28], 8 RCTs [8,9,12,13,19,21,24,25], 1 cohort [14], 5 quasi-experimental [9,16,17,20,29], and 1 case series [27]	Level 1a	Available evidence demonstrates a positive effect of MFT in reducing self-reported sleepiness and increasing QoL.
Is MFT useful for treating SDB? (snoring)	1 meta-analysis [15], 1 systematic review [28], 7 RCTs [8,11,12,19,24,25,26,29], 6 quasi-experimental [9,16,17,18,20]	Level 1a	Available evidence demonstrates a positive effect of MFT in reducing snoring as measured by objective (PSG) and subjective (scales) evaluation.
Is MFT useful for treating upper airway resistance syndrome?	None	None	There is no evidence regarding the use of MFT for upper airway resistance syndrome.
Which patients benefit most from MFT?	1 RCT [11] and 1 quasi-experimental [17]	None	There is no evidence regarding the optimal method for patient selection for MFT.
What are the secondary effects of MFT?	1 meta-analysis [10], 3 RCTs [8,11,12,13,19,21,24,25,26], and 1 cohort [14]	Level 1b	Available evidence suggests that MFT is a safe therapy.
Which type of MFT is best?	1 RCT [11]	Level 2b	Available evidence has important flaws, and there is no evidence for making solid conclusions.
How well do patients adhere to MFT?	9 RCTs [8,11,12,13,19,21,24,25,26], 7 quasi-experimental [9,16,17,18,20,22,29], and 1 case series [27]	Not applicable	Several variables can influence adherence to MFT. Available evidence is too heterogeneous to make solid conclusions about adherence.
How long do the effects of MFT last?	1 RCT [21] and 1 case series [27]	Not applicable	There is no evidence.

MFT—myofunctional therapy; SDB—sleep-disordered breathing; PSG—polysomnographic; RCT—randomized controlled trial; QoL—quality of life.

## Data Availability

Not applicable.

## Supplementary Materials

The following are available online at https://www.mdpi.com/article/10.3390/ijerph18147291/s1. Table S1: Search strategy. Table S2: Summary of included studies; Table S3: Myofunctional therapy protocols.
