# Supplementary material for: The Role of Myofunctional Therapy in Treating Sleep-Disordered Breathing: A State-of-the-Art Review
Source: Int J Environ Res Public Health. 2021 Jul 8;18(14):7291. doi: 10.3390/ijerph18147291 (PMC8306407; doi:10.3390/ijerph18147291)
Supplement: Supplementary file 1 [file ijerph-18-07291-s001.zip › ijerph-1285928-supplementary.pdf]

Table S1. Search strategy.

Database	Search strategy	References
Pubmed	(((((Apnea[Title/Abstract]) OR (Apnoea[Title/Abstract])) OR (Snoring[Title/Abstract])) OR ("disordered breathing"[Title/Abstract])) OR (OSA[Title/Abstract])) AND (((((((Myofunction*[Title/Abstract]) OR ("tongue exercis*"[Title/Abstract])) OR ("oropharyngeal exercis*"[Title/Abstract])) OR ("myofascial reeducation"[Title/Abstract])) OR ("Speech therapy"[Title/Abstract])) OR ("Airway exercise"[Title/Abstract])) OR ("tongue-muscle training"[Title/Abstract]))	110
Cochrane Library	#1(Apnea):ti,ab<br>#2 (Apnoea):ti,ab<br>#3 (Snoring):ti,ab<br>#4 ("disordered breathing"):ti,ab<br>#5 (OSA):ti,ab<br>#6 (Myofunction*):ti,ab<br>#7 ("tongue exercis*"):ti,ab<br>#8 ("oropharyngeal exercis*”):ti,ab<br>#9 ("myofascial reeducation"):ti,ab<br>#10 ("Speech therapy"):ti,ab<br>#11 ("Airway exercise"):ti,ab<br>#12 ("tongue-muscle training"):ti,ab<br><br>(#1 OR #2 OR #3 OR #4 OR #5) AND (#6 OR #7 OR #8 OR #9 OR #10 OR #11 OR #12)	38
EMBASE	((Apnea or Apnoea* or Snoring or "disordered breathing" or OSA) and (Myofunction* or "tongue exercis*" or "oropharyngeal exercis*” or "myofascial reeducation" or "Speech therapy" or "Airway exercise" or "tongue-muscle training")).ti	76
Scopus	(TITLE-ABS (Apnea) OR TITLE-ABS (Apnoea*) OR TITLE-ABS (Snoring) OR TITLE-ABS ("disordered breathing") OR TITLE-ABS (OSA)) AND (TITLE-ABS (Myofunction*) OR TITLE-ABS ("tongue exercis*") OR TITLE-ABS ("oropharyngeal exercis*”) OR TITLE-ABS ("myofascial reeducation") OR TITLE-ABS ("Speech therapy") OR TITLE-ABS ("Airway exercise") TITLE-ABS ("tongue-muscle training"))	42
SciELO	((Apnea) OR (Apnoea*) OR (Snoring) OR ("disordered breathing") OR (OSA)) AND ((Myofunction*) OR ("tongue exercis*") OR ("oropharyngeal exercis*) OR ("myofascial reeducation") OR ("Speech therapy") OR ("Airway exercise") OR ("tongue-muscle training"))	11

Bibliography of selected papers	Hand search	4
Total		281
Total After duplicates removal		192

Table S2. Summary of included studies.

Author (year)	Design	Sample size	Population mean ± SD (range)	Inclusion criteria	Exclusion criteria	Follow up after MFT	Adherence (%)	PSG variables assessed	Clinical variables assessed
Atilgan E et al 2020	Open RCT	Experimental: 15	50.40 ± 13.49 (NR)	Mild to moderate OSA (AHI 5-30) by PSG	Hyperthyroidism. Previous stroke history. Neuromuscular disease. Severe obstructive nasal disease	NR	100%	None	PSQI. ESS. SF-36. BDI. 6MWT.
		Control: 15	52.26 ± 17.39 (NR)						
Suzuki M et al 2020	Quasi-experimental	40	69.3 ± 1.5 (NR)	Moderate to severe OSA using CPAP. Older than 44 years	BMI ≥ 30. Craniofacial malformation. Neuromuscular disease. Previous stroke. Heart failure. Severe obstructive nasal disease	NR	80%	AHI. Average apnea duration. REM. AHI. LSAT. T90. Snore proportion.	Tongue strength pressure. ESS. BMI.
Hsu B et al 2020	Metanalysis	394	NR ± NR (NR)	RCT on patients with OSA	NR	NR	NR	AHI. AI. LSAT. Sleep efficiency.	ESS. PSQI. Snoring frequency. Snoring intensity.
Erturk et al 2020	Open RCT	MFT: 18	53.71 ± 7.08 (NR)	Mild, moderate, or severe OSAS not using CPAP	Stroke. Neurological disease. Severe obstructive nasal disease. History of infection within the last month. BMI ≥ 40.	NR	77.78%	AHI. Sleep efficiency.	ESS. Berlin questionnaire. FOSQ. PSQI. FSS.
		Inspiratory training: 18	49.66 ± 9.08 (NR)				83.34%		
		Placebo: 18	47.25 ± 7.32 (NR)				66.67%		
Kim J et al 2020	Open RCT	Experimental group: 16	53.88 ± 18.44 (NR)	Adults <18 years old. AHI>5. Non-compliant with CPAP	Severe obstructive nasal disease. Craniofacial malformation. Neuromuscular disease. Hypothyroidism. Severe comorbidities. Experience playing woodwind instruments, or singers.	NR	82.06 ± 23.70%	AHI. LSAT. Snoring intensity. Snoring frequency.	ESS. PSQI. Dry mouth. Intensity of snoring (VAS).
		Control group: 15	49.20 ± 19.40 (NR)				72.52 ± 30.09%		
O'Connor-Reina et al 2020 (Nov)	Single blinded RCT	Experimental: 18	59.17 ± NR (NR)	Newly diagnosed severe OSA	BMI ≥ 40. Inability to complete the questionnaires. Drug or alcohol abuse. Hypnotic medication. Uncontrolled coronary disease. Decompensated heart failure. Previous stroke. Systemic disease associated with an inflammatory-related entity. Neuromuscular disease. Craniofacial deformity. Active oncology. Previous use of MFT or other treatments for sleep apnea. Severe upper airway obstruction. Tongue-tie. Temporomandibular joint disorder.	NR	90%	IAH. ODI.	ESS. PSQI. IOPI (Lip and Tongue).
		Control: 10	63.9 ± NR (NR)				NA		
O'Connor et al 2020 (Feb)	Prospective cohort study	Experimental: 15	36 ± 13.5 (NR)	OSAS AHI>5	Patients with previous OSA surgery. Upper airway malformation.	NR	75%	AHI. LSAT.	ESS

		Control: 5	53.2 ± 7.7 (NR)				NA		
Diaz M et al 2019	Case series	12	65 ± 9.03 (52-82)	Adults. Mild OSA (AHI 5-15). Moderate OSA (AHĺ 15-30) not tolerant to CPAP.	No compliant with MFT. Uncomplete data in the clinical record.	3-6 months	79.8	Total sleep time. Sleep efficiency. Sleep latency. REM sleep latency. AHI. LSAT. Arousal index. REM sleep time. N3 sleep time.	ESS. Neck circumference.
Goswami U et al 2018	Open RCT	Experimental: 8	51 ± 11 (NR)	20-65 years. BMI ≤ 32. Habitual snorers with AHI 0– 14.	Comorbid sleep disorders. (including excessive daytime sleepiness with ESS > 11). Significant medical comorbidities including decompensated cardiopulmonary disease and chronic rhinitis. Alcohol or opioid use. Using CPAP. > 5% weight change. Patients with a less than 10 Mb/s wifi connection where they sleep. Inability to sleep in a quiet environment, or a loud snorer as a bed partner.	NR	50%	Snoring	Subjective snoring
		Controls: 8	51 ± 10 (NR)				50%		
Camacho M et al 2018	Metanalysis	211	NR ± NR (NR)	Snoring. MFT as sole treatment.	Studies with additional treatments performed. Studies using devices. Studies without data for MFT alone.	NR	NR	Snoring frequency. Snoring intensity.	Bedpartner VAS. Snoring severity. Berlin questionnaire.
Kayamori F et al 2017	Systematic review	NR	NR ± NR (NR)	Portuguese or English. Study of MFT in SBD. Reporting PSG data (pre and post therapy).	NR	NR	NR	AHI. LSAT.	ESS. Berlin questionnaire.
Verma RK et al 2016	Quasi-experimental	34	41.1 ± 10.6 (26–61)	AHI>5 and <30 confirmed by PSG	BMI ≥ 40. Craniofacial malformations. Regular use of hypnotic medications. Hypothyroidism. Previous stroke. Neuromuscular disease. Heart failure. Coronary disease. Severe obstructive nasal disease	NR	58.8%	AHI. ODI. Arousal index. % Sleep stages. Sleep efficiency. LSAT. Mean SaO2. T90.	Neck circumference. ESS. Morning headache. Witnessed apnea. BMI. Snoring intensity. Berlin questionnaire.
Mohamed AS et al 2016	Quasi-experimental	Moderate OSA: 15	46.39 ± 2.04	AHI>15 with at least two symptoms of OSA (snoring, fragmented sleep, witnessed apneas, morning headache or daytime sleepiness)	> 50 years old. BMI ≥40. Current or planned intervention for weight reduction. Craniofacial malformations. Physical obstruction in nose or throat. Abnormally large tonsils. Any neurological or psychiatric disease. Regular use of alcohol/drugs known to affect sleep or daytime sleepiness. Hypothyroidism. Previous stroke. Neuromuscular disease. Heart failure. Coronary disease.	NR	83.3%	AHI. ODI. T90. Snoring index. LSAT.	ESS. Neck circumference. BMI.
		Severe OSA: 15	47.5 ± 9						
Nemati S et al 2015	Quasi-experimental	63	45.35 ± 10.08 (22–65)	Snoring > 1 year. Age 20-65.	Inability to learn the exercises. <80% compliance rate. Sedative medications.	1 Week	84.12%	None	SSS. Snoring (VAS score). Subjective

					Severe OSAS. BMI ≥ 40. Advanced pulmonary diseases. Craniofacial malformations. Hypothyroidism. Cerebrovascular accident. Rhinosinusitis. Neuromuscular diseases. Heart failure. Coronary artery disease. Severe obstructive nasal diseases. Tonsillitis.				assessment of snoring frequency and severity.
Ieto V et al 2015	Single blinded RCT	Experimental: 19<br>Control: 20	48 ± 14 (NR)<br>45 ± 13 (NR)	Recent diagnosis of primary snoring or mild to moderate OSA. Age 20-65.	BMI ≥40. Smokers. Alcohol abuse. Edentulous. Severe nasal obstruction. Tonsils grade 3-4. Craniofacial malformations. Regular use of hypnotic medications. Severe comorbidities.	NR	85 ± 8%<br>NA	AHI. Sleep efficiency. LSAT. O2 desaturation index. Snore index.	Subjective snoring (intensity, frequency). PSQI. ESS.
		Controls: 9							
Camacho M et al 2015	Metanalysis	Adults: 120<br>Children: 25	44.5 ± 11.6 (NR)<br>8.4 ± 3.1 (NR)	Peer-reviewed studies evaluating isolated MFT for either adult or pediatric OSA. Reporting PSG data, snoring, and/or sleepiness data.	Studies evaluating singing or instrument playing. Studies without quantitative data. Studies in which the MFT patients also underwent additional interventions. If individual patient data were reported and patients lost 10% or more of their body weight, then those patients were excluded.	NR	NR	AHI. ODI. Snoring.<br>AHI	ESS. Subjective assessment of snoring<br>None
Matsumura E et al 2014	Quasi-experimental study	11	55.1 ± NR (39-72)	Mild to severe OSAS or primary snoring with recent PSG (<3m)	BMI ≥ 40. Regular use of psychotropic medication. Use of CPAP. Hypothyroidism. Previous stroke. Cardiac or neuromuscular disorders. Severe nasal obstruction. Craniofacial malformation. Temporomandibular dysfunction. Severe systemic disease. Previous surgical procedures to correct OSA.	NR	81.8%	None	Adapted Berlin questionnaire. ESS.
Diaféria G et al 2013	Single blinded RCT	MFT: 27<br>CPAP: 27	45.2 ± 13.0 (NR)<br>46.4 ± 9.1 (NR)	OSAS confirmed with PSG, Men. BMI<35	Female. Uncooperative, illiterate, or who had a low education level. Other sleep disorders or previous treatment for OSA. Serious or decompensated clinical or psychiatric medical illnesses. Patients who use alcohol, stimulants or sedatives. Grade	3 Months	63%<br>30%	Arousal index. AHI. LSAT.	ESS. FOSQ. WHOQOL. SF 36.

		MFT and CPAP: 22	47.5 ± 10.9 (NR)		III or IV palatine tonsils. Grade II or III septal deviation. Evident micrognathia.		65%		
		Control: 24	42.9 ± 10.5 (NR)				55%		
Suzuki H et al 2013	Quasi-experimental study	6	22 ± 0.5 (22-23)	Students with ESS>10 and mild to moderate OSA	Systemic illness. Stomatognathic abnormalities	NR	100%	AHI. Mean SaO2.	None
Baz H et al 2012	Quasi-experimental study	30	44.07 ± 7.54 (NR)	Mild to moderate OSA	BMI > 40. Craniofacial malformation. Using hypnotics. Hypothyroidism. Previous stroke. Neuromuscular disease. Heart failure. Coronary artery disease. Severe OSA. Physical obstruction in nose or throat. Abnormally large tonsils. Deviated septum. Drug/alcohol abuse. Depression. Previous treatment for snoring.	NR	75%	AHI. ODI. T90. LSAT. Arousal index. % total sleep time snoring.	Snoring. ESS. Morning headache. Nocturnal choking. Witnessed apnea.
Valbuza JS et al 2010	Systematic Review of RCT	Case: 86 Controls: NR	NR± NR (NR)	Any clinical criteria for OSA	Studies including patients with obstruction in the nose or throat. Abnormally large tonsils. Deviated septum. Drug/alcohol abuse. Smoking. Depression. Previous treatment for OSA. Neurological or psychiatric disorders. Pregnancy or lactation. Use of drugs acting on central nervous/neuromuscular system. Diabetes mellitus. Serious cardiac arrhythmias. Wearing a pacemaker, cardioverter or defibrillator. Trauma and cutaneous lesions.	NR	NR	AHI	ESS. PQSI. Partner rating of sleep disturbance. Attention test. FOSQ. SF-36. Modified Berlin Questionnaire.
Guimarães KC et al 2009	Single blinded RCT	Experimental: 16	51.5 ± 6.8 (NR)	25–65-year-old. Recent diagnosis of mild to moderate OSA	BMI ≥ 40. Craniofacial malformations. Regular use of hypnotic medications. Hypothyroidism. Previous stroke. Neuromuscular disease. Heart failure. Coronary disease. Severe obstructive nasal disease.	NR	84,2%	Sleep efficiency. AHI in REM and NREM. AI. HI.	Berlin Questionnaire. ESS. PSQI.
		Control: 15	47.7 ± 9.8 (NR)				75%		
Randerath WJ et al 2004	Double blinded RCT	Experimental: 33	50.8 ± 12.1 (NR)	OSAS with AHI 10-40. Adults (19-75 year old)	Previous treatment for OSA. Acute heart failure. Serious cardiac arrhythmia. Other acute diseases necessitating immediate	NR	97.06%	AHI. LSAT. Total sleep time. Snoring. Sleep in S3-4. Total REM sleep.	Attention test. ESS. FOSQ. Self-assessment

	Control: 34	53.3 ± 11.3 (NR)	treatment with CPAP. Neurologic or psychiatric disorders. Pregnancy or lactation. Use of drugs acting on the neuromuscular system. Wearing a pacemaker, cardioverter or defibrillator. Insulin dependent diabetes. Trauma, cutaneous lesions, or prior surgery in the submental region.	72.73%	Wake after sleep onset. RAI. Arousals.	questionnaire of side effects.

NR (not reported) / NA (not applicable) / RCT (randomized clinical trial) / VAS (visual analogue scale) / PSG (polysomonography) / AT (adenotonsillectomy) / OSA (obstructive sleep apnea / LSAT (lowest oxygen saturation) / AHI (Apnea-Hypopnea index) / AI (apnea index) / HI (hypopnea index) / T90 (total sleep time with oxygen saturation) / BMI (body mass index) / CPAP (continuous positive airway pressure) / RDI (respiratory disturbance index) / RERA (respiratory event related arousal) / IOPI (Iowa oral performance instrument) / ADHD (attention deficit hyperactive disorder) / FSS (Fatigue severity scale) / RAI (respiratory arousal index)/ SF-36 (Short Form 36) / ESS (Epworth Sleepiness scale) / FOSQ (Functional Outcome of Sleep Questionnaire) / PSQI (Pittsburgh sleep questionnaire Index) / SCR (Sleep Clinical Record) / SSS (snoring scale score) / BDI (Beck Depression Inventory) / 6MWT (Six Minute Walk Test) / IPAQ-SF (International Physical Activity Short Form) / (WHOQoL) World Health Organization Quality of Life

Table S3. Myofunctional therapy protocols. NR (not reported) / PSG (polysomonography)

Author (year)	Exercises	Duration of the therapy (months)	Sessions	Passive / active MFT	At-home / supervised MFT
Atilgan E et al 2020	(number and specific exercises not explained. Based on Guimarães et al 2019)<br>Tongue, jaw and swallowing exercises	3	Sessions: 7 days a week.4 times a day.<br>Length: maximum 30 min	Active	1 weekly visit supervised by a physiotherapist
Erturk N et al 2020	17 exercises. Soft palate (2 exercises). Tongue (4 exercises), Facial muscles (8 exercises). Stomatognathic (2 exercises). Chewing and swallowing (1 exercise). (based on Guimarães et al 2009)	3	Sessions: once a day. 5 days a week.<br>Length: NR	Active	5 Training sessions with speech therapist. Then at home exercises.
Suzuki M et al 2020	4 exercises. Tongue (1 exercise). Check (1 exercise). Palate (1 exercise). Nasal breathing rehabilitation (1 exercise).	6	Sessions: 3 times/day<br>Length: NR	Active	At home supervised
Kim J et al 2020	10 exercises. Tongue and lip exercises (modification of the Stanford protocol)	4	Sessions: 10 times/day.<br>Length: 10 repetitions each exercise	Active	At home, two groups supervised with coaching and unsupervised
O'Connor-Reina C et al 2020 (Nov)	Airway Gym app. Proprioceptive rehabilitation (tongue, cheek, neck muscles with acoustic and visual feedback).	3	Sessions: 1 session a day<br>Length: 20 min	Active	At home exercises. Supervised by telemedicine
O'Connor Reina C et al 2020 (Feb)	Airway Gym app. Proprioceptive rehabilitation (tongue, cheek, neck muscles with acoustic and visual feedback).	3	Sessions: 1 session a day<br>Length: 20 min	Active	At home exercises. Supervised by telemedicine
Diaz M et al 2019	6 exercises: Tongue (4 exercises), buccinator and lips (1 exercise), soft palate (1 exercise)	NR	Sessions: 3 sessions a day<br>Length: 5-10 min	Active	At home with clinical follow up.

					1-2 weekly follow up.
Goswami U et al 2018	3 exercises of vowel vocalization and articulation.	3	Sessions: daily<br>Length: 15 min	Active	At home. Follow up not reported.
Verma RK et al 2016	Different exercises that changed every month to increase difficulty and strength (lip, tongue, jaw, palate)<br>Phase 1-13 exercises: 4 lip exercises, 5 tongue exercises, 2 jaw exercises, and 2 soft palate exercises.<br>Phase 2-13 exercises: 2 lip exercises, 2 tongue exercises, 2 jaw exercises, 5 soft palate exercises, and 2 cheek exercises.<br>Phase 3-7 exercises: 2 lip exercises, 2 tongue exercises, 1 jaw exercise, and 2 soft palate exercises.	3	Sessions: 5 times a day<br>Length: 10 times each exercise	Active	At home. Weekly supervised by a speech therapist.
Mohamed AS et al 2016	5 exercises: Tongue (3 exercises), palate, buccinator (1 exercise) and chewing exercises (1 exercise)	3	Sessions: 3-5 times a day<br>Length: 10 min	Active	At home. Weekly supervised by a speech therapist.
Nemati S et al 2015	10 exercises (modified from Guimarães et al): Soft palate (2 exercises), tongue exercises (4 exercises), facial exercises (4 exercises). Following a program in a compact disc.	3	Sessions: 5 times a week<br>Length: 30 minutes.	Active	Initial visit with speech therapist. 1 visit every 3-4 weeks.
Ieto V et al 2015	7 exercises: Tongue (3 exercises), soft palate (2 exercise), cheek (2 exercises).	3	Sessions: 3 times a day<br>Length: 8 minutes	Active	Weekly visits to speech therapist to evaluate adherence. Exercises performed at home.
Matsumura E et al 2014	14 exercises (based on Guimarães et al 2009): Soft palate (2 exercises), tongue (4 exercises), facial (5 exercises). Stomatognathics function (2 exercises). Swallowing and Chewing (1 exercise).	NR	Sessions: 12 sessions. Frequency NR.<br>Length: 40 min	Active	Session with a speech therapist

Diaféria G et al 2013	14 exercises: Soft palate (2 exercises), tongue (4 exercises), facial (5 exercises). Stomatognathic function (2 exercises). Swallowing and Chewing (1 exercise). (based on Guimarães et al 2009)	3	Sessions: 3 times a day. Daily exercise. Length: 20 min each session.	Active	Weekly therapist visit. Exercises performed at home.
Suzuki H et al 2013	Exercises with Patakara Lip trainer	2	Sessions: 2 a day, 4 days/week Length: 5 min	Active	Supervised by speech therapist
Baz H et al 2012	11 exercises: tongue (9 exercises), palate (2 exercises) (Adapted from Guimaraes et al and Galye Burditt)	3	Sessions: 3-5 times a day Length: 10 minutes	Active	Twice a week supervised. Then exercises performed at home.
Guimarães KC et al 2009	14 exercises: Soft palate (2 exercises), tongue (4 exercises), facial (5 exercises). Stomatognathics function (2 exercises). Swallowing and Chewing (1 exercise).	3	Sessions: NR Length: 30 min a day	Active	Instructed by a speck therapist. Weekly supervised. Exercises at home.
Randerath WJ et al 2004	Stimulation device (Apone-Stim 400 Muscle Stimulator, BMR Neurotech Ltd., Derrybeg Industrial Estate, Co. Donegal, Ireland)	2	Sessions: 2 a day Length: 20 minutes	Passive	At home. Telephone supervised.
